# German translation, cultural adaptation, and validation of the Health Literacy Questionnaire (HLQ)

**DOI:** 10.1371/journal.pone.0172340

**Published:** 2017-02-24

**Authors:** Sandra Nolte, Richard H. Osborne, Sarah Dwinger, Gerald R. Elsworth, Melanie L. Conrad, Matthias Rose, Martin Härter, Jörg Dirmaier, Jördis M. Zill

**Affiliations:** 1 Department of Psychosomatic Medicine, Center for Internal Medicine and Dermatology, Charité—Universitätsmedizin Berlin, Berlin, Germany; 2 Population Health Strategic Research Centre, School of Health and Social Development, Deakin University, Geelong, Victoria, Australia; 3 Department of Medical Psychology, University Medical Center Hamburg-Eppendorf, Hamburg, Germany; 4 Quantitative Health Sciences, Outcomes Measurement Science, University of Massachusetts Medical School, Worcester, Massachusetts, United States of America; Yokohama City University, JAPAN

## Abstract

The Health Literacy Questionnaire (HLQ), developed in Australia in 2012 using a ‘validity-driven’ approach, has been rapidly adopted and is being applied in many countries and languages. It is a multidimensional measure comprising nine distinct domains that may be used for surveys, needs assessment, evaluation and outcomes assessment as well as for informing service improvement and the development of interventions. The aim of this paper is to describe the German translation of the HLQ and to present the results of the validation of the culturally adapted version. The HLQ comprises 44 items, which were translated and culturally adapted to the German context. This study uses data collected from a sample of 1,058 persons with chronic conditions. Statistical analyses include descriptive and confirmatory factor analyses. In one-factor congeneric models, all scales demonstrated good fit after few model adjustments. In a single, highly restrictive nine-factor model (no cross-loadings, no correlated errors) replication of the original English-language version was achieved with fit indices and psychometric properties similar to the original HLQ. Reliability for all scales was excellent, with a Cronbach’s Alpha of at least 0.77. High to very high correlations between some HLQ factors were observed, suggesting that higher order factors may be present. Our rigorous development and validation protocol, as well as strict adaptation processes, have generated a remarkable reproduction of the HLQ in German. The results of this validation provide evidence that the HLQ is robust and can be recommended for use in German-speaking populations.

**Trial Registration**: German Clinical Trial Registration (DRKS): DRKS00000584. Registered 23 March 2011.

## 1. Introduction

Health literacy describes the personal motivation and ability to access, appraise, and use health information to judge and decide upon healthcare (both preventive and health promoting measures) [1]. This concept has been increasingly recognised over the past decade [2, 3] due to growing evidence that many health inequalities and health outcomes are closely linked to individuals’ health literacy levels [4]. For example, low health literacy levels have been associated with poor adherence to medications [5], lower use of preventive healthcare services [6], increased hospitalisations [7], and increased mortality [8]. While research suggests clear links between health literacy and health outcomes, a major shortcoming of these findings is that the data were frequently derived from suboptimal measurement instruments. For example, Jordan *et al*. (2011) found salient limitations regarding the general conceptualisation of health literacy coupled with weak psychometric properties of the instruments used [9].

Due to these shortcomings, the Health Literacy Questionnaire (HLQ) was developed in Australia in 2012 [10]. In order to ensure that the HLQ included variables that are pertinent to all stakeholders involved, particularly community members, patients and clinicians, the developers of the HLQ applied a comprehensive process across all stages of instrument development, validation, and implementation, a process they had previously introduced as ‘the validity-driven approach’ [11]. This method has already been applied successfully across several instrument development projects [12–14]. An important feature of the process is the deliberate avoidance of theory when conceptualising the target construct. Instead, a broad range of stakeholders is invited to brainstorm ideas on a defined topic in interviews and concept mapping workshops. Concept mapping workshops are a structured approach to systematically generate ideas, which are then organised into clusters, analysed, and finally grouped into a ‘concept map’ [15, 16]. Concept mapping facilitates the emergence of a theoretical model that is grounded in the daily lives and practices of community members, patients, and practitioners. The statements and clusters generated during this process form the basis for the questions (items) and the latent variables (domains/scales) of the new questionnaire [15].

For the development of the HLQ, the consultation process included two concept mapping workshops as well as interviews with patients in Australia, and two nominal group workshops with both patients and experts overseas. These exercises and several steps of item writing and testing in both a calibration (n = 634) and a validation (n = 405) sample eventually led to the final instrument consisting of nine distinct health literacy domains. Applying a strict confirmatory factor model (no cross-loadings, no correlated residuals) resulted in excellent psychometric properties of the final instrument [10]. In addition to its strong psychometric properties, the particular strengths of the HLQ lie in its practical relevance. That is, the nine HLQ domains have been developed in a way that they can be used not only in standard surveys and outcomes assessments but to also derive health literacy profiles, which in turn can facilitate intervention development, service improvement, and evaluation. Among other projects in Australia and overseas, these profiles are currently applied in the Ophelia (OPtimising HEalth LIteracy and Access) Study [4, 17].

To date, there is no health literacy instrument in Germany with comparable content, application, and psychometric properties as have been described for the HLQ. While the European Health Literacy Survey is a large and important project [18], including a German translation of the European Health Literacy Survey Questionnaire (HLS-EU-Q) [19], it was designed to compare populations [18] instead of facilitating intervention development, service improvement, and evaluation as is intended by the HLQ [10]. Further, while a head-to-head comparison between the two instruments is still missing, the face validity of the HLS-EU-Q suggests only some overlap between the two instruments. Hence, the HLQ and the HLS-EU-Q may rather complement each other, depending on the purpose of the (research) project being undertaken. Further, the Functional Communicative Critical Health Literacy (FCCHL) questionnaire from Japan was recently translated into German. Given its brevity, however, the translators critically discussed that it may be insufficient to capture the entire concept of health literacy [20].

As a result, the HLQ was deemed an important measure of health literacy for German-speaking populations, and it was therefore translated and culturally adapted to the German context. The aim of this study was to investigate the psychometric properties of the German HLQ (HLQ-G).

## 2. Methods

### 2.1. The Health Literacy Questionnaire

The HLQ consists of 44 items forming nine domains of health literacy [10]. The scales are:

‘1 Feeling understood and supported by healthcare providers’ (4 items),
‘2 Having sufficient information to manage my health’ (4 items),
‘3 Actively managing my health’ (5 items),
‘4 Social support for health’ (5 items),
‘5 Appraisal of health information’ (5 items),
‘6 Ability to actively engage with healthcare providers’ (5 items),
‘7 Navigating the healthcare system’ (6 items),
‘8 Ability to find good health information’ (5 items), and
‘9 Understanding health information well enough to know what to do’ (5 items).

The first five scales are scored on a 4-point Likert-type response scale (*Strongly disagree*, *Disagree*, *Agree*, *Strongly agree*), later referred to as ‘Part I items’. The latter four scales are scored on a 5-point response scale where respondents rate the items by level of difficulty in undertaking a task (ranging from *Cannot do* to *Very easy*), later referred to as ‘Part II items’.

### 2.2. Translation and cultural adaptation

The translation and cultural adaptation of the HLQ followed a standardised protocol provided by the authors of the original instrument [21]. This included: a forward translation by a professional, registered translator as mandated in the protocol (German-English bilingual, native German speaker) who followed an item intent guide; critical review by the German research team (SN, JMZ); and a blinded independent back translation (German-English bilingual, native English speaker; MLC). Apart from the translation, items were reviewed for cultural appropriateness and measurement equivalence (i.e., each concept in the German version was as ‘strong’ as the original English version). The meaning of every nuance in the final translation was verified with one of the original authors (RHO) against all previous language versions through written reports and a consensus conference.

### 2.3. Setting

Data for the validation of the HLQ-G were collected as part of a large-scale randomised controlled trial (RCT) that was aimed at assessing the effectiveness of a telephone-based health coaching (TBHC) intervention. Inclusion criteria for the RCT were: insuree of the KKH (Kaufmännische Krankenkasse Hannover), a large German health insurance company, age (≥18 years), sufficient German language skills, ability to participate in telephone coaching, and presence of at least one chronic condition at the time of study entry. Included diagnoses were: diabetes, coronary artery disease, asthma, hypertension, heart failure, chronic obstructive pulmonary disease, chronic depression or schizophrenia. The RCT was carried out by the University Medical Center Hamburg-Eppendorf, Germany, over a period of four years, with data collected at baseline (T_0_), 12 (T_1_), 24 (T_2_), and 36 (T_3_) months. Further details on the RCT are described elsewhere [22].

Study participants were grouped into intervention group, controls, and people who had declined to participate. As the RCT had already begun at the time of the HLQ translation, the HLQ-G could only be included at T_3_. Given that the intervention group had already been exposed to the TBHC at that point, variation in these data was deemed too low to be suitable for a validation sample, which was supported by preliminary analyses of these data. Consequently, we decided to exclude intervention group subjects from the validation. T_3_ sample sizes were n = 580 (controls) and n = 494 (declined participation) leading to a sample size of n = 1,074.

Ethics approval was obtained from the Ethics Committee of the State Chamber of Physicians in Hamburg (Germany). All participants gave written informed content.

### 2.4. Missing data and data analyses

For data preparation, cases that had missed more than half of the HLQ-G items were excluded, leading to the elimination of 16 cases. Of the remaining cases, each HLQ-G item was missed by any average of 2.6% of the respondents. Item 8_1, *Find information about health problems* had the largest amount of missing data (5.5%). Since no missing data patterns were apparent, data were deemed to be missing completely at random (MCAR) [23]. Missing data were replaced by the expectation-maximisation (EM) algorithm [24], which was carried out in LISREL’s data pre-processor program PRELIS [25]. The final sample size available for the current analyses was n = 1,058. The dataset is available in supporting information S1 File S1_HLQ-G_data.sav.

As the purpose of this study was the validation of the German translation of the HLQ, our analyses were confirmatory by nature. First, we tested for internal consistency of each HLQ scale by calculating Cronbach’s Alpha (α). Second, confirmatory factor analyses (CFA) were carried out using polychoric correlations and the asymptotic covariance matrix as input matrices to accommodate the ordinal scaling of the items. For this, we first fitted nine one-factor congeneric models to more closely explore items that were conceptualised as belonging to the same HLQ factor. Potential weak loadings and/or correlated residuals were noted for the next step. In this next step, the full nine-factor model was fitted to the data defined as a strict CFA, i.e. neither cross-loadings nor correlated errors were allowed. Robust Maximum Likelihood was used for parameter estimation.

To evaluate model fit, we applied the fit indices Root Mean Square Error of Approximation (RMSEA), Non-Normed Fit Index (NNFI)/Tucker Lewis Index (TLI), Comparative Fit Index (CFI), and the Standardised Root Mean Residual (SRMR). For the RMSEA, a value of ≤0.05 was interpreted as close fit, while values of ≤0.08 were interpreted as acceptable fit [26]. For both NNFI/TLI and CFI a cut-off value of ≥0.95 was applied, while for the SRMR a value of ≤0.08 was deemed appropriate [27, 28].

Statistical analyses were carried out using IBM SPSS Statistics version 22 (for the characterisation of the sample and Cronbach’s Alpha) and LISREL version 8.72 (for CFA).

## 3. Results

### 3.1. Results of the translation and cultural adaptation

After receiving the forward translation from the professional translator, the German research team accepted the recommended translation of only three (of 23) Part I items, while the remaining translations of the Part I items and all translations of the 21 Part II items were challenged. Of these, 11 Part I items and 17 Part II items were simplified to better match lower literacy levels. Efforts were made to balance overall item similarity to the original wording, while ensuring equivalent item intent and cultural appropriateness for the German context. Of the remaining items (nine Part I items; four Part II items), only minor adjustments were undertaken to achieve satisfactory wording. For example, the term for ‘health professional’ in German (i.e., ‘Fachkräfte im Gesundheitswesen’) is not as common in German as the term is used in English, and the team also perceived the term as potentially confusing for future users of the HLQ-G. Therefore, the term ‘doctors and therapists’ (i.e., ‘Ärzte und Therapeuten’) was adopted from the translation of the Health Education Impact Questionnaire (heiQ) [29].

The working translation was discussed with the original professional forward translator until a solution was found that was agreeable amongst the entire translator team. After back translation of the final translation, an 8-hour consensus teleconference was carried out with the Australian lead (RHO) and the German research team involved in either forward or back translation (SN, MLC, JMZ).

### 3.2. Demographic characteristics of the sample

As shown in Table 1, no significant differences were observed between the TBHC control group and those who had declined TBHC participation. Slightly more women than men participated in the study (56.5% women, 43.5% men); age ranged between 22 and 87 years with a mean age of 71.1 (SD 8.0) years. The majority of participants were between 70 to 74 and 75 to 79 years of age, 28.7% and 31.0% respectively. Almost three quarters (72.7%) of respondents were living with a partner. Just under half of the sample had nine years or less of schooling, while 7.9% had a university degree (data not shown). The vast majority of respondents were retired (76.7%). The breakdown of net monthly household income was as follows: up to €1,000 (18.2%), €1,001 and €1,500 (25.2%), €1,501 and €2,000 (23.7%), €2,001 and €2,500 (18.3%), €2,501 or more (14.5%).

**Table 1:** Sociodemographic characteristics of the sample; total sample and comparison of control group (n = 570) versus those who declined participation (n = 488).

	Total		Control		Declined	
	sample		group		participation	
	n = 1,058		n = 570		n = 488	
	n	(%)	n	(%)	n	(%)
Gender						
Male	453	(43.5)	242	(42.9)	211	(44.1)
Female	589	(56.5)	322	(57.1)	267	(55.9)
Age in years						
Mean (SD)	71.1 (8.0)		71.3 (7.9)		71.0 (8.2)	
Range	22–87		35–86		22–87	
Age groups						
up to 60 years	93	(8.9)	50	(8.9)	43	(9.0)
60–64 years	108	(10.4)	55	(9.8)	53	(11.1)
65–69 years	125	(12.0)	77	(13.7)	48	(10.1)
70–74 years	299	(28.7)	151	(26.8)	148	(31.0)
75–79 years	323	(31.0)	180	(31.9)	143	(30.0)
80+ years	93	(8.9)	51	(9.0)	42	(8.8)
Relationship status						
Living with partner	688	(72.7)	357	(70.1)	331	(75.6)
Living alone	259	(27.3)	152	(29.9)	107	(24.4)
Years of schooling						
Year 9 or less	486	(48.5)	267	(49.0)	219	(47.8)
Year 10	269	(26.8)	141	(25.9)	128	(27.9)
>10 years	248	(24.7)	137	(25.1)	111	(24.2)
Employment status						
Part-/full-time	84	(8.5)	48	(8.9)	36	(8.1)
Early retirement	52	(5.3)	33	(6.1)	19	(4.3)
Retired	754	(76.7)	411	(76.0)	343	(77.6)
Unemployed	38	(3.9)	19	(3.5)	19	(4.3)
Other	55	(5.6)	30	(5.5)	25	(5.7)
Net income						
Up to €1,000	170	(18.2)	96	(18.7)	74	(17.6)
€1,001 to €1,500	236	(25.2)	116	(22.6)	120	(28.5)
€1,501 to €2,000	222	(23.7)	122	(23.7)	100	(23.8)
€2,001 to €2,500	171	(18.3)	101	(19.6)	70	(16.6)
€2,501 or more	136	(14.5)	79	(15.4)	57	(13.5)

Note: No significant differences were found between the control group and those who declined participation (chi-square tests; for age in years: t-test statistic for independent samples; p<0.05).

### 3.3. Internal consistency of the nine HLQ factors

Internal consistency of all HLQ scales was high, with a Cronbach’s Alpha of at least 0.77. In detail, these were: α = 0.86 for ‘1. Feeling understood and supported by healthcare providers’, α = 0.81 for ‘2. Having sufficient information to manage my health’, α = 0.78 for ‘3. Actively managing my health’, α = 0.79 for ‘4. Social support for health’, α = 0.77 for ‘5. Appraisal of health information’, α = 0.91 for ‘6. Ability to actively engage with healthcare providers’, α = 0.87 for ‘7. Navigating the healthcare system’, α = 0.88 for ‘8. Ability to find good health information’, and α = 0.84 for ‘9. Understanding health information well enough to know what to do’.

### 3.4. Confirmatory factor analysis

To investigate whether the previously established factor structure was tenable for the HLQ- G, nine one-factor models were fitted to the data first. The fit statistics of the one-factor congeneric models suggested largely better fit than those of the English-language HLQ [10]. In detail, seven factors did not need any further model adjustment as sufficiently satisfactory, while two factors required one adjustment each (correlated error) to obtain satisfactory model fit.

In a second step, the full nine-factor model was fitted to the data. Applying a strict confirmatory factor model, i.e. not allowing for cross-loadings or correlated errors, the factor structure of the original HLQ was fully replicated with fit indices similar to the original English-language version. The fit indices of the full factor model of the HLQ-G were: χ^2^_SB_(866) = 2948.1, p<0.000; RMSEA = 0.048 (90% CI, 0.046;0.050); NNFI = 0.99; CFI = 0.99; SRMR = 0.075.

As shown in Table 2, factor loadings of the HLQ-G items in each of the nine scales were satisfactory to high, ranging between 0.61 and 0.94, with 38 out of the 44 HLQ items showing a factor loading of ≥0.70. About 75% of the factor loadings were remarkably similar to the original HLQ (deviations of ≤0.10), with most English items showing marginally higher loadings. The largest deviations, where respective factor loading of either the German- or the English-language version was <0.70, were seen in item 3_1 (*I spend quite a lot of time actively managing my health*, German loading: 0.66; loading of the original HLQ: 0.79), item 4_2 (*When I feel ill*, *the people around me really understand what I am going through*, German: 0.61; original: 0.83), item 7_2 (*Get to see the healthcare providers I need to*, German: 0.84; original: 0.61), and item 9_2 (*Accurately follow the instructions from healthcare providers*, German: 0.69; original: 0.82).

**Table 2:** Factor loadings of the nine-factor model of the Health Literacy Questionnaire in German (HLQ-G); Cronbach’s Alpha (α) by individual HLQ scale.

Scale / item	Part I scales					Part II scales			
	1	2	3	4	5	6	7	8	9
1_1	.87								
1_2	.94								
1_3	.74								
1_4	.92								
‘1. Feeling understood and supported by healthcare providers’: α = 0.86									
2_1		.71							
2_2		.85							
2_3		.88							
2_4		.87							
‘2. Having sufficient information to manage my health’: α = 0.81									
3_1			.66						
3_2			.76						
3_3			.80						
3_4			.74						
3_5			.72						
‘3. Actively managing my health’: α = 0.78									
4_1				.70					
4_2				.61					
4_3				.85					
4_4				.67					
4_5				.86					
‘4. Social support for health’: α = 0.79									
5_1					.71				
5_2					.70				
5_3					.80				
5_4					.63				
5_5					.64				
‘5. Appraisal of health information’: α = 0.77									
6_1						.83			
6_2						.89			
6_3						.89			
6_4						.86			
6_5						.88			
‘6. Ability to actively engage with healthcare providers’: α = 0.91									
7_1							.74		
7_2							.84		
7_3							.82		
7_4							.81		
7_5							.73		
7_6							.76		
‘7. Navigating the healthcare system’: α = 0.87									
8_1								.82	
8_2								.86	
8_3								.84	
8_4								.83	
8_5								.78	
‘8. Ability to find good health information’: α = 0.88									
9_1									.80
9_2									.69
9_3									.87
9_4									.72
9_5									.80
‘9. Understanding health information well enough to know what to do’: α = 0.84									

Fit indices of the replication of the full 9-factor HLQ model in German (HLQ-G): χ^2^_SB_(866) = 2948.1, p<0.000; RMSEA = 0.048; NNFI = 0.99; CFI = 0.99; SRMR = 0.075.

### 3.5. Factor correlations

As shown in Table 3, inter-factor correlations between the nine HLQ factors ranged from 0.34 to 0.96. While all Part I scales showed small- to medium-size correlations, some very large inter-factor correlations were found between Part II scales. Three correlations were around 0.9: HLQ scales ‘6. Ability to actively engage with healthcare providers’ and ‘7. Navigating the healthcare system’ (correlation: 0.96), HLQ scales 7. and ‘8. Ability to find good health information’ (correlation: 0.90), and HLQ scales 8. and ‘9. Understanding health information well enough to know what to do’ (correlation: 0.92).

**Table 3:** Factor correlations of the nine factors of the HLQ-G.

Scale	Part I scales					Part II scales		
	1	2	3	4	5	6	7	8
2	.640							
3	.474	.696						
4	.614	.586	.532					
5	.347	.579	.756	.408				
6	.685	.669	.423	.517	.343			
7	.618	.711	.459	.466	.343	.957		
8	.473	.740	.463	.385	.512	.829	.897	
9	.440	.633	.417	.344	.374	.827	.880	.917

For the names of the HLQ scales, see Table 2.

## 4. Discussion

This study undertook rigorous processes to translate and culturally adapt the Health Literacy Questionnaire to the German context, and to validate the translated version. The data indicate excellent reproduction of the original English-language HLQ items in a different language, culture, and healthcare setting. The HLQ-G has satisfactory psychometric properties, suggesting that this instrument measures nine distinct domains of health literacy [10]. All scales also demonstrated excellent reliability. In summary, the results from this study, using data from a large RCT on TBHC [22], suggest an almost perfect replication of the original HLQ and its hypothesised factor structure.

The vast majority of respective HLQ-G factor loadings were well within the range of the original HLQ loadings, with over 75% of the items showing deviations of 0.10 or less. Items with larger deviations in respective factor loading are difficult to interpret in the context of translations of self-report instruments. One could argue that respective latent variable has less influence on responses on items that showed a smaller loading, following Bollen’s (1989) notion of the “direct structural relation” (p.197) between latent and indicator variable [30]. As smaller loadings were more frequently observed for HLQ-G items, it can be interpreted as the respective item being less important to define its hypothesised latent variable in the German-language version. However, as we are dealing with a translation and cultural adaptation of a self-report instrument–as opposed to a revalidation of an instrument in the same language–it is also conceivable that the meaning of respective latent variable and/or the individual item is slightly different in the two cultures.

Further, the Part 1 item response scale may be perceived differently between the English and the German version. When comparing the HLQ with the heiQ [12, 29], a questionnaire that some of the authors developed and translated several years ago, the 4-point response scale contained *slightly disagree* and *slightly agree* as the mid categories. As *disagree*/*agree* is comparatively strong in German as used in the present HLQ, adding *slightly* may have been more suitable for the HLQ in the German context. Before such a change could be made, however, this question would need to be tested thoroughly. A change in response format compared to the original English-language HLQ would not only severely compromise the comparability between English and German HLQ data but it would affect the many translations of the HLQ that are currently underway, such as Danish, Dutch, and Norwegian. It remains to be shown whether this observation is also an issue in other translation efforts.

Our study has some limitations. First, we used a convenience sample from a large RCT on TBHC recruited from a health insurance company. The sample not only included insurees of that particular company, but also respondents with at least one chronic condition. Further, mean age was high (71 years), the level of education was relatively low (48.5% reported ≤9 years of schooling), and 76.7% indicated being retired; hence, the sample recruited for the coaching intervention was not representative of the German general population. The HLQ is aimed to be implemented across populations, for example, as a screening instrument. Consequently, further work needs to be done in a wider range of respondents, such as the calibration sample of the original HLQ in Australia consisting of a wide range of respondents from community health, hospital, and home care settings [10]. However, given that the German sample was older with rather low educational background is reassuring at the same time. That is, given the particular context of our study, i.e. health literacy, this first validation of the HLQ-G was undertaken in a sample that may be expected to have more problems with this type of questionnaire. Therefore, we feel the sample does not weaken the study but it may indeed strengthen it. That is, it is expected that it should be possible to replicate our findings in alternative samples that are more representative of the German general population, i.e. samples that would be younger with a higher educational status. In summary, these first results show promising psychometric properties of the translated version of the HLQ, which was based on a large sample ensuring robustness of the statistical outputs. However, it remains that these results should be confirmed in the general population as well as in different disease groups.

Second, our experience suggests that use of a professional translator may not be necessary, indeed even potentially counterproductive. Registered translators often focus on generating a linguistically accurate translation, rather than ensuring readability by people who may have a limited vocabulary or ensuring that the actual meaning of each item is conveyed. This appeared to be the case in our study where careful re-consideration of the item intent by the translation team resulted in at least some adjustment to over 90% of the initial forward translations. For future translation endeavours, we suggest that it would be easier and more efficient to have the translations carried out by bilingual field workers, psychometricians or researchers from related fields with experience in instrument translations.

Third, the finding of high to very high inter-factor correlations of Part II factors requires further work. While neither the item content of each scale nor the statistical output suggest combining factors at this stage, alternative model specifications, for example with higher order factors or a bifactor solution, should be explored in future research. This includes Bayesian Structural Equation Modeling, which may be a more appropriate approach for these kinds of data as it allows for some ‘wiggle room’ for factor loadings and residual correlations [31]. That is, a model as strict as the one applied may impose unnecessarily narrow restrictions on the model, which may have led to an artificial inflation of the inter-factor correlations [32].

## 5. Conclusion

In conclusion, our results demonstrate that the HLQ was successfully translated, culturally adapted, and the original robust psychometric properties reproduced in German. Strong psychometric properties including excellent reliability and good fit statistics of the HLQ-G show that the original English-language version was well replicated. We recommend our translation of the Health Literacy Questionnaire for use in German-speaking populations to reliably assess people’s health literacy levels.

## Supporting information

S1 File S1_HLQ-G_data.sav. (SAV)
